# Protective Effects of L-Carnitine on Intestinal Ischemia/Reperfusion Injury in a Rat Model

**DOI:** 10.4021/jocmr540w

**Published:** 2011-04-04

**Authors:** Yong Yuan, Hao Guo, Yi Zhang, Dong Zhou, Ping Gan, Dao Ming Liang, Jia Yong Chen

**Affiliations:** a Department of Emergency, The Second Affiliated Hospital of Kunming Medical College, Kunming, Yunnan 650101, China; b Department of Cardiology, Calmette Hospital, Kunming Medical College, Kunming, Yunnan 650011, China

## Abstract

**Background:**

Ischemia/reperfusion (IR) injury of the intestine is a major problem in abdominal pathological condition and is associated with a high morbidity and mortality. The purpose of the study is to determine whether the L-carnitine can prevent the harmful effects of small intestinal IR injury in rats.

**Methods:**

Thirty Sprague-Dawley rats were randomly divided into three groups. Sham operated group (S), for shamoperated, the IR group for rats submitted to 45-minute of intestinal ischemia and 2-hour reperfusion, and IR+L group for those IR group treated with L-carnitine before reperfusion. All the rats were given EmGFP labelled E. coli DH5α through gavage 2-hour before the operative procedure. Afterwards the bacterial translocation (BT) from mesenteric lymph nodes (MLN), liver, spleen, lung and portal vein blood were detected. And the colony forming units/g (CFU/g) were counted. The TNF-α, IL-1β, IL-6, and IL-10 in serum were measured by ELISA. The morphometric study was measured by Chius classification.

**Results:**

The levels of BT were higher in the IR group than IR+L group (P < 0.05). The E. coli DH5α was hardly detected in the S group. The IR+L rats had enhancement of IL-10 and suppressed production of serum TNF-α, IL-1β and IL-6, compared to IR group rats (P < 0.05). The degree of pathological impairment in small intestine was lighter in IR+L than IR group (P < 0.05).

**Conclusions:**

The L-carnitine pretreatment has a positive effect on reducing levels of BT, on inhibiting secretion of proinflammatory cytokines, and on lessening intestinal mucosa injury during small intestinal IR injury.

**Keywords:**

L-carnitine; Ischemia/reperfusion injury; Intestine

## Introduction

The gastrointestinal tract is a tissue which is highly sensitive to ischemia-reperfusion (IR) injury in the body [1]. The intestinal IR injury is caused by many clinical conditions, including acute mesenteric ischemia, intestinal obstruction, incarcerated hernia, small intestine transplantation, neonatal necrotizing enterocolitis, trauma, and shock [2-4]. It is extremely dangerous and emergent for patients to have intestinal IR injury because it may result in an array of severe clinical syndromes, and even death [5]. Under the normal physiological situation, the maintenance of bacteria and their products in the intestine depends on mucosal mucin and a layer of epithelial cells, and the unbroken intestinal barrier is essential for health and survival. In addition, the epithelial cells have high metabolism activity, and it is also susceptible to oxygen deprivation with subsequent ischemia damage to enterocytes and their supporting structures [6]. The impairment of the intestinal barrier may lead to decrease in absorption of nutrition, and the bacteria in the intestine may translocate to blood and other organs due to the damage of the intestinal barrier. The consequences of the bacterial translocation (BT) and release of harmful substances produced by the bacteria into blood circulation are to activate inflammatory mediators and initiate Systemic Inflammatory Response (SIRS) or Multiorgan Dysfunction Syndrome (MODS) [7, 8]. Thus, there is a great interest in exploring methods to protect the small intestine from lesions. L-carnitine is an important active material, which plays a crucial role in energy metabolism. The shortage of L-carnitine includes metabolism disruption and induction of a series of disorders. Xie et als study has demonstrated that L-carnitine has a protective effect on IR injury, which is partly due to its prevention of energy loss and its antioxidant activity [9]. Moreover, it has a protective effect on oxidative stress-induced DNA damage [10]. Oyanagi et als study [11] has demonstrated that L-carnitine can maintain mitochondrial function and suppress oleic acid-mediated membrane permeability transition (MPT) of mitochondria through acceleration of beta-oxidation. The opening of MPT has been proved as a key event in cell apoptosis or necrosis after IR injury [12, 13]. It has been suggested that L-carnitine would prevent apoptosis and maintain functions of cell. The purpose of this experiment is to explore the effects of L-carnitine on the rats suffering from the small intestinal IR injury through detecting translocation of EmGFP labelled E. coli from the mesenteric lymph nodes, liver, spleen and portal vein blood. Additionally, the small intestinal morphometric study was performed and the levels of serum cytokines (TNF-α, IL-1β, IL-6 and IL-10) were investigated.

## Materials and Methods

### Animals

A total of thirty adult SD rats were obtained from the Experimental Research Center of Zhengzhou University, and their weight is in the range of 280 - 320 g. All of the rats were maintained under standard conditions with controlled temperature (18 ± 4 ^o^C), 12 hours light and 12 hours darkness cycle, and feeding with standard food and water. The maintenance of the experimental rats was administrated by licensed operators and these rats were given humane treatment. This study was approved by the Ethical Committee of Kunming Medical University.

### Labeling of Escherichia coli

**Figure 1.**
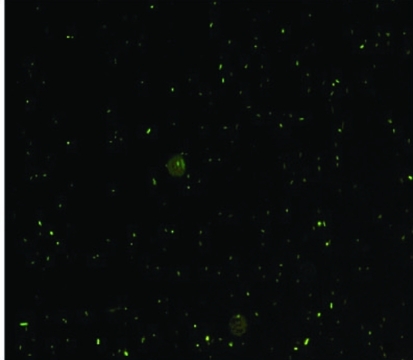
The E. coli with GFP were detected with fluorescence microscope. The cells displayed rod shape and erupted fluorescent light.

The Escherichia coli DH5α (donated from PHD Cao, the Department of Micro-organism of Kunming Medical University) was selected to label with EmGFP. The brief procedures are as follows. 1. Establishment of competent bacteria using CaCl_2_ method. 2. Transformation of plasmid DNA (pRSET-EmGFP bought from the Invitrogen Company) into E. coli using the heat shock method. A mixture of the competent bacteria and the plasmid DNA were placed in iced bath for 30 minutes and put at 42 ^o^C for 2 minutes (heat shock), and then placed back into the iced bath. After transformation, the bacteria were cultured on Luria-Bertani plate with ampicillin (100 mg/L) at 37 ^o^C for 12 hours. Four colonies were inoculated into liquid media (200 ml) and grown at 37 ^o^C with agitation for 16 hours in order to ensure growth of isolated colonies. The E. coli DH5α with transformed plasmid containing EmGFP were detected by using fluorescence microscope (Fig. 1). The bacteria were cultured for the study to the density of 7.5 10^6^/ml.

### Intestinal IR injury model and operative procedures

The thirty healthy SD rats were randomly divided into three groups (n = 10 for each group): sham operated group (S), IR group (IR) and IR+L-carnitine group (IR+L). All of the animals were given the EmGFP labeled E. coli 2 ml by gavage two hours before surgical procedures. The rats were anesthetized through injecting Urethane 200 mg/kg into the peritoneal cavity. In the S group, the rats received a 3-centimeter medium-length-wise laparotomy, and the small intestine was exposed. Subsequently, the superior mesenteric artery (SMA) was identified and dissected, and then the peritoneal cavity was closed. In the IR group, the rats underwent intestinal ischemia for 45 minutes through occlusion of the SMA with a micro vascular clamp. The occluding clamp was removed after ischemia for a reperfusion period for 2 hours. In the IR+L group, the rats were given intervention of intestinal ischemia, which was the same as that in the IR group, and they were injected L-caritine 80 mg/kg suspended in saline solution (total volume = 2 ml) through tail vein before reperfusion injury. Meanwhile, the rats were given 2 ml saline solution instead of L-caritine in both the S and IR groups.

**Table 1:** The Chiu’s Score Classification of Small Intestinal Injury

Classification	Pathological change
Level 0	Mucosa without changes.
Level 1	Well-constituted villosities, no cellular lysis or inflammatory process, although there is formation of Grunhagen’s sub-epithelial space.
Level 2	Presence of cellular lysis, formation of Grunhagen’s sub-epithelial space and increased spacing among the villosities.
Level 3	Destruction of the free villosities section, presence of dilated capillaries and inflamed cells.
Level 4	Structural destruction of the villosities, only traces of some villosities, formed by inflamed cells and necrotic material, with hemorrhage and basal glandular ulceration.
Level 5	Destruction of all the mucosa, no glandular structure can be seen, only the amorphous material laying on the sub-mucosa tissue.

At the end of the procedures, one milliliter venous systemic serum was collected in order to measure the levels of TNF-α, IL-1β, IL-6 and IL-10 in venous blood using ELISA. The rats were sacrificed and a one-centimeter segment of ileum was dissected and processed using standard histological techniques including formalin fixation, dehydration and paraffin embedding, and was subsequently cut into 4 m sections and stained with Haematoxylin and Eosin. All of the sections were analyzed with an optic microscope by the pathologist who did not know which group each rat belonged to, and they were classified according to the degree of tissue injury in accordance to Chiu’s score classification [14] (Table 1). The homogenate of the mesenteric lymph nodes, liver, spleen, lung and half milliliter blood of portal vein were detected for the BT. Among the homogenate, 0.1 gram was cultured in agar culture media with ampicillin (100 mg/L) at 37 ^o^C for 24 hours in order to count the CFU/g of bacteria.

### Statistical analysis

Data was presented as mean ± standard derivation (SD). One-way analysis of variance (ANOVA) and Tukey Post Hoc (parametric) test were used to compare serum cytokines and translocation of bacteria between the three groups. Histopathologic grades of each group were compared using Kruskal-Wallis test and Tukey Post Hoc (nonparametric) test. The significance level was established in P < 0.05 for all the tests.

## Results

### The levels of BT in different groups

**Table 2:** The Levels of BT From MLN, Liver, Lung, Spleen and Portal Vein Blood (CFU/g) (Mean ± SD)

Groups	n	MLN	Liver	Lung	Spleen	Portal vein blood
S	10	1.2 ± 1.03^Δ^	0.0 ± 0.00^Δ^	0.0 ± 0.00^Δ^	0.0 ± 0.00^Δ^	2.0± 2.58^Δ^
IR	10	25.1 ± 3.41*	20.5 ± 3.03*	25.5 ± 3.92*	15.2 ± 3.01*	19.9 ± 3.70*
IR+L	10	11.3 ± 2.36	10.0 ± 2.58	10.8 ± 2.25	10.3 ± 3.43	9.3 ± 1.64

± P < 0.05 compared to IR and IR+L groups * P < 0.05 compared to IR+L group

The levels of BT had a significant increase in animals distributed to the IR group, compared to animals in the IR+L-carnitine and S groups (P < 0.05). In the S group, there were few colony forming units in culture plates of MLN and portal vein blood (Table 2).

### Changes of intestinal mucosa under light microscope

**Figure 2.**
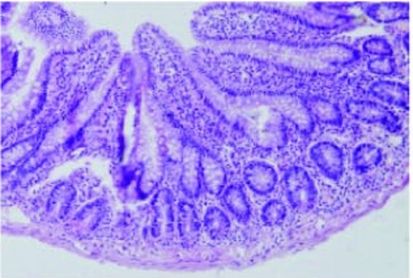
Normal intestinal mucosa in the S group rats (Grade 0) (H&E, 100).

**Figure 3.**
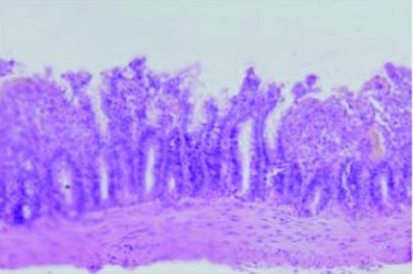
Intestinal villus after IR in group II rats, with lifted epithelium, white blood cells, and monocytes in the Lamina propria (Grade 4) (H&E, × 100).

**Figure 4.**
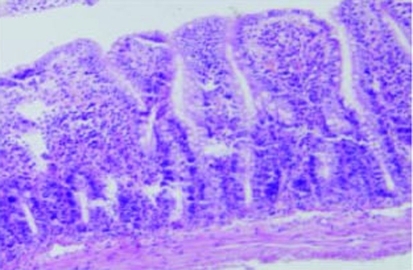
Development of Gruenhagen’s space at tip of a villus in group III rats (Grade 2) (H&E, × 100).

The villus and glands were normal and inflammatory cell infiltration was not observed in mucosal epithelial layer in the sham operated group (Fig. 2). Multiple erosions, inflammatory cells and bleeding were observed in the IR group (Fig. 3). Lighter edema of mucosa villus and fewer infiltration of necrotic epithelial inflammatory cells neutrophil leukomonocyte were found in mucosa epithelial layer in IR+L group than the IR group (Fig. 4).

### Chiu’s score of small intestinal structure

**Table 3:** The Levels of Chiu’s Score in Small Intestinal Structure (Mean ± SD)

Groups	n	Small intestinal structure
S	10	0 ± 0.00^Δ^
IR	10	4.0 ± 0.41*
IR+L	10	2.4 ± 0.46

Δ P < 0.05 compared to IR and IR+L groups * P < 0.05 compared to IR+L group

In all of the three groups, the Chius score was highest in the IR group, and it was lowest in the S group (P < 0.05). The score in the IR+L group was significantly lower than that in the IR group (P < 0.05) (Table 3).

### Changes in TNF-α and interleukins in vena systemic serum

**Table 4:** The Levels of TNF-α and Interleukins in Arterial Serum (pg/ml)

Groups	n	TNF-α	IL-1	IL-6	>IL-10
S	10	53.82 ± 8.94^Δ^	195.52 ± 52.26^Δ^	259.89 ± 89.41^Δ^	359.38 ± 76.32^Δ^
IR	10	575.02 ± 50.56*	601.29 ± 58.33*	567.25 ± 69.62*	126.06 ± 50.37*
IR+L	10	104.86 ± 10.61	291.58 ± 76.88	258.75 ± 77.58	254.65 ± 67.91

Δ P < 0.05 compared to IR and IR+L groups * P < 0.05 compared to IR+L group

The concentrations of pro-inflammatory factors including TNF-α, IL-1, and IL-6 in the experimental rats’ serum in the IR and IR+L group were higher than that in the S group (P < 0.05). And the levels of TNF-α, IL-1 and IL-6 in the IR group rats were higher than that in the IR+L group (P < 0.05). The levels of inhibitive inflammatory factor IL-10 in the IR+L group were lower than that in the IR group (P < 0.05) (Table 4).

## Discussion

The primary functions of the intestine are to absorb nutrients and exclude food debris, bacteria and their products. The maintenance of these functions relies on the integrity of mucosal and barrier of intestine. However, under certain pathological circumstances such as intestinal IR injury, the intestine may lose its barrier function so that the systemic microbial infection, and even MODS could be induced [7]. Compared to other internal organs, the intestine is the organ that is most sensitive to IR injury in the body [15, 16]. There is increasing evidence from experimental and clinical studies to support the ‘intestinal hypothesis of sepsis’, which is based on bacterial or endotoxin translocation from the intestinal lumen to the extra-intestinal sites [17]. Nevertheless, growing acceptance of the ‘intestinal hypothesis of sepsis’ was mainly triggered by indirect supports rather than concrete scientific evidence.

IR injury of the intestine is a complex, multifactorial, and pathophysiological process that involves the dysfunction of absorption, BT, actions of oxygen-derived free radicals, cytokines, nitric oxide, and PMNs [18]. IR injury to the small intestine causes local production of the ROS, which is known to play an important role in gut epithelial damage [19]. Intestinal mucosal mast cells (IMMC) are particularly frequent in close proximity to epithelial surfaces, where they are strategically located for optimal interaction with the environment and for their putative functions of host defense [20]. Previous studies have demonstrated that the degranulation of IMMC can be induced by oxidants generated in the post-ischemic gut, and the released inflammatory mediators such as histamine and tumor necrosis factor-α (TNF-α) could aggravate the injury to intestine after reperfusion [21, 22]. In line with general IR-induced cell damage, also in intestinal IR, apoptosis is the major mode of cell death in the destruction of epithelial cells [23].

The intestinal IR injury can lead to mucosal cells energy exhaustion and is also associated with decrease in ATP synthesis [24-26]. Mitochondrium is one of the most important organelle and regarded as a “dynamic factory”, and it is vulnerable to injury due to IR. Undoubtedly, the damage of mitochondria will induce energy exhaustion and failure of maintenance of cellular basic functions. The defective mitochondrial oxidative phosphorylation and metabolic compromise play an important role in IR injury [27]. Increased ROS changes the mitochondrial membrane permeability and results in opening of mitochondrial permeability transition (MPT) [28]. The opening of MPT is a key event in cell death after ischemia-reperfusion [12, 13, 29]. Opening of the nonspecific mitochondrial permeability transition pore (mPTP) in the inner mitochondrial membrane results in the collapse of the membrane potential (Δψm), uncoupling of the respiratory chain, and efflux of cytochrome c and other proapoptotic factors that may lead to either apoptosis or necrosis [30]. Opening of the mPTP is triggered by Ca^2+^ overload and excessive production of ROS in the early minutes of reflow, which is crucial event in reperfusion injury [31, 32]. Griffiths and Halestrap’s study [33] have demonstrated that in isolated rat heart, the mPTP remained closure throughout ischemia but opened at the time of reperfusion.

L-Carnitine can carry long-chain fatty acylgroups into mitochondria for beta-oxidation. And it acts as a scavenger of oxygen free radical, relief of oxidative stress, and reduction of lipid peroxidation [34]. Therefore, L-Carnitine plays a protective role in metabolic disorders. Virmani and Gaetanis study [35] revealed that L-carnitine could maintain the MPT and modulate the activation of the mitochondrial permeability transition pores (mPTP), especially the cyclosporin-dependent mPTP. Furthermore, their preliminary experiments also showed that LC may reduce the peroxynitrite levels and protect against the underlying mechanism of methamphetamine toxicity [36].

In this study, the hypothesis that enhancement of mitochondrial energy metabolism by using L-carnitine could prevent the generation of inflammatory factors and protect the integrality of mucosal barrier was tested. Moreover, the levels of TNF-α, IL-1β, IL-6, and IL-10 in serum (Table 4). The translocation of the E. Coli DH5α from extra-intestinal sites was examined in the study (Table 2) in order to obtain direct support for using L-carnitine that protects the intestinal barrier.

In conclusion, the increase of L-carnitine density in IR intestine is a protective event. Mucosal injury and BT were reduced in the L-carnitine group, compared with the IR group (P less than 0.05). And L-carnitine effectively inhibited releasing of pro-inflammatory factors, compared with IR group (P less than 0.05). In addition, L-carnitine can protect intestine from IR injury because it can improve absorption of nutrients and prevention of BT. Nevertheless, clinical investigations will be required in order to confirm the effectiveness of L-carnitine as a therapeutic agent in clinical practice.

## References

[1] Mojzis J, Hviscova K, Germanova D, Bukovicova D, Mirossay L (2001). Protective effect of quercetin on ischemia/reperfusion-induced gastric mucosal injury in rats. Physiol Res.

[2] Mallick IH, Yang W, Winslet MC, Seifalian AM (2004). Ischemia-reperfusion injury of the intestine and protective strategies against injury. Dig Dis Sci.

[3] Guneli E, Cavdar Z, Islekel H, Sarioglu S, Erbayraktar S, Kiray M, Sokmen S (2007). Erythropoietin protects the intestine against ischemia/ reperfusion injury in rats. Mol Med.

[4] Teke Z, Kabay B, Aytekin FO, Yenisey C, Demirkan NC, Sacar M, Erdem E (2007). Pyrrolidine dithiocarbamate prevents 60 minutes of warm mesenteric ischemia/reperfusion injury in rats. Am J Surg.

[5] Aldemir M, Gurel A, Buyukbayram H, Tacyildiz I (2003). The effects of glucose-insulin-potassium solution and BN 52021 in intestinal ischemia-reperfusion injury. Vasc Endovascular Surg.

[6] Grotz MR, Ding J, Guo W, Huang Q, Deitch EA (1995). Comparison of plasma cytokine levels in rats subjected to superior mesenteric artery occlusion or hemorrhagic shock. Shock.

[7] Heckbert SR, Vedder NB, Hoffman W, Winn RK, Hudson LD, Jurkovich GJ, Copass MK (1998). Outcome after hemorrhagic shock in trauma patients. J Trauma.

[8] Antonsson JB, Fiddian-Green RG (1991). The role of the gut in shock and multiple system organ failure. Eur J Surg.

[9] Xie J, Zeng Q, Wang L (2006). The protective effect of L-carnitine on ischemia-reperfusion heart. J Huazhong Univ Sci Technolog Med Sci.

[10] Berni A, Meschini R, Filippi S, Palitti F, De Amicis A, Chessa L (2008). L-carnitine enhances resistance to oxidative stress by reducing DNA damage in Ataxia telangiectasia cells. Mutat Res.

[11] Oyanagi E, Yano H, Kato Y, Fujita H, Utsumi K, Sasaki J (2008). L-Carnitine suppresses oleic acid-induced membrane permeability transition of mitochondria. Cell Biochem Funct.

[12] Duchen MR, McGuinness O, Brown LA, Crompton M (1993). On the involvement of a cyclosporin A sensitive mitochondrial pore in myocardial reperfusion injury. Cardiovasc Res.

[13] Griffiths EJ, Halestrap AP (1993). Protection by Cyclosporin A of ischemia/reperfusion-induced damage in isolated rat hearts. J Mol Cell Cardiol.

[14] Chiu CJ, McArdle AH, Brown R, Scott HJ, Gurd FN (1970). Intestinal mucosal lesion in low-flow states. I. A morphological, hemodynamic, and metabolic reappraisal. Arch Surg.

[15] Granger DN, Hollwarth ME, Parks DA (1986). Ischemia-reperfusion injury: role of oxygen-derived free radicals. Acta Physiol Scand Suppl.

[16] Yamamoto S, Tanabe M, Wakabayashi G, Shimazu M, Matsumoto K, Kitajima M (2001). The role of tumor necrosis factor-alpha and interleukin-1beta in ischemia-reperfusion injury of the rat small intestine. J Surg Res.

[17] Ceppa EP, Fuh KC, Bulkley GB (2003). Mesenteric hemodynamic response to circulatory shock. Curr Opin Crit Care.

[18] Carden DL, Granger DN (2000). Pathophysiology of ischaemia-reperfusion injury. J Pathol.

[19] Park PO, Haglund U, Bulkley GB, Falt K (1990). The sequence of development of intestinal tissue injury after strangulation ischemia and reperfusion. Surgery.

[20] Penissi AB, Rudolph MI, Piezzi RS (2003). Role of mast cells in gastrointestinal mucosal defense. Biocell.

[21] Andoh A, Fujiyama Y, Araki Y, Kimura T, Tsujikawa T, Bamba T (2001). Role of complement activation and mast cell degranulation in the pathogenesis of rapid intestinal ischemia/reperfusion injury in rats. Digestion.

[22] Boros M, Ordogh B, Kaszaki J, Nagy S (1999). The role of mast cell degranulation in ischaemia-reperfusion-induced mucosal injury in the small intestine. Ann Acad Med Singapore.

[23] Ikeda H, Suzuki Y, Suzuki M, Koike M, Tamura J, Tong J, Nomura M (1998). Apoptosis is a major mode of cell death caused by ischaemia and ischaemia/reperfusion injury to the rat intestinal epithelium. Gut.

[24] Kuenzler KA, Pearson PY, Schwartz MZ (2002). Interleukin-11 enhances intestinal absorptive function after ischemia-reperfusion injury. J Pediatr Surg.

[25] Prasad R, Alavi K, Schwartz MZ (2001). GLP-2alpha accelerates recovery of mucosal absorptive function after intestinal ischemia/reperfusion. J Pediatr Surg.

[26] Rajeevprasad R, Alavi K, Schwartz MZ (2000). Glucagonlike peptide-2 analogue enhances intestinal mucosal mass and absorptive function after ischemia-reperfusion injury. J Pediatr Surg.

[27] Jassem W, Fuggle SV, Rela M, Koo DD, Heaton ND (2002). The role of mitochondria in ischemia/reperfusion injury. Transplantation.

[28] Tsujimoto Y, Shimizu S (2007). Role of the mitochondrial membrane permeability transition in cell death. Apoptosis.

[29] Nazareth W, Yafei N, Crompton M (1991). Inhibition of anoxia-induced injury in heart myocytes by cyclosporin A. J Mol Cell Cardiol.

[30] Bernardi P, Petronilli V (1996). The permeability transition pore as a mitochondrial calcium release channel: a critical appraisal. J Bioenerg Biomembr.

[31] Piper HM, Meuter K, Schafer C (2003). Cellular mechanisms of ischemia-reperfusion injury. Ann Thorac Surg.

[32] Di Lisa F, Menabo R, Canton M, Barile M, Bernardi P (2001). Opening of the mitochondrial permeability transition pore causes depletion of mitochondrial and cytosolic NAD+ and is a causative event in the death of myocytes in postischemic reperfusion of the heart. J Biol Chem.

[33] Griffiths EJ, Halestrap AP (1995). Mitochondrial non-specific pores remain closed during cardiac ischaemia, but open upon reperfusion. Biochem J.

[34] Augustyniak A, Skrzydlewska E (2009). L-Carnitine in the lipid and protein protection against ethanol-induced oxidative stress. Alcohol.

[35] Virmani A, Gaetani F, Imam S, Binienda Z, Ali S (2003). Possible mechanism for the neuroprotective effects of L-carnitine on methamphetamine-evoked neurotoxicity. Ann N Y Acad Sci.

[36] Virmani A, Gaetani F, Imam S, Binienda Z, Ali S (2002). The protective role of L-carnitine against neurotoxicity evoked by drug of abuse, methamphetamine, could be related to mitochondrial dysfunction. Ann N Y Acad Sci.

